# HRL-Det: Hierarchical Reinforcement Learning for Sequential Object Detection in Aerial Imagery

**DOI:** 10.3390/s26134232

**Published:** 2026-07-03

**Authors:** Meng Li, Yaowen Hu

**Affiliations:** 1 College of Geography and Environment, Xianyang Normal University, Xianyang 712000, China; 2 School of Computer Science, National University of Defense Technology (NUDT), Changsha 410073, China; yaowenhu@nudt.edu.cn

**Keywords:** object detection, unmanned aerial vehicle, reinforcement learning, hierarchical reinforcement learning, neural ordinary differential equation, Lyapunov stability, reward shaping, deep Q-network

## Abstract

Object detection in unmanned aerial vehicle (UAV) imagery suffers from severe scale variation, dense object packing, and prohibitive computational cost when conventional detectors exhaustively evaluate high-resolution frames. Reinforcement learning (RL)-based sequential detectors offer a promising alternative by formulating localization as an active search process, yet existing methods are limited by discrete-time state transitions, sparse reward signals, and premature policy collapse. In this paper, we propose HRL-Det, a hierarchical reinforcement learning framework that addresses these challenges through two tightly coupled innovations. First, a Neural ODE-driven Continuous-Time Bellman State Evolution module models the agent’s state dynamics as a stochastic differential equation governed by the Hamilton–Jacobi–Bellman equation, enabling fine-grained temporal reasoning with memory-efficient adjoint-based backpropagation. Second, a Lyapunov-Guided Entropy-Regularized Reward Shaping mechanism constructs convergence-promoting dense rewards informed by Lyapunov stability analysis while maintaining exploration diversity through maximum entropy optimization. Extensive experiments on VisDrone2019, DroneVehicle, and MS COCO 2017 show that HRL-Det achieves mAP@0.5 of 0.412, 0.812, and 0.735, respectively, outperforming existing RL-based detectors and achieving competitive accuracy relative to representative non-RL detectors under the same COCO metric, while requiring only 17.3 M parameters and an average of 6.3 search steps per object.

## 1. Introduction

Object detection in unmanned aerial vehicle (UAV) imagery is a fundamental and challenging task in computer vision, aiming to identify and localize objects of interest from an aerial perspective. It has been widely applied in many real-world scenarios, such as intelligent traffic monitoring [1], emergency search and rescue [2], power line inspection [3], and railway infrastructure surveillance [4]. Due to the high altitude of flight and the complexity of ground environments, improving the detection accuracy and operational efficiency of drones remains an important and challenging research topic [5,6].

In recent years, deep learning-based object detection methods have achieved remarkable progress. Existing detectors can be roughly divided into three categories. Two-stage detectors, such as Faster R-CNN [7] and Cascade R-CNN [8], first generate region proposals and then perform refinement. One-stage detectors, such as the YOLO series [9,10] and SSD [11], directly predict object categories and locations from feature maps for real-time inference. More recently, transformer-based and anchor-free detectors, such as Swin Transformer [12] and CenterNet [13], have further improved performance by modeling long-range dependencies or eliminating predefined anchor constraints.

Although existing deep learning-based detectors have achieved impressive performance, they still face significant limitations in UAV scenarios. Conventional methods usually rely on dense prediction or exhaustive window sliding, which introduces high computational costs when processing high-resolution aerial images on resource-constrained edge devices [14]. Furthermore, these static architectures often suffer from a “performance cliff” when detecting tiny objects in complex backgrounds, leading to high false-positive rates and significant resource waste on irrelevant background regions [15,16]. Therefore, designing an efficient framework that balances precision and computational overhead is crucial.

To overcome the inefficiency of dense prediction, reinforcement learning-based (RL) object detection has emerged as a promising technical route. Unlike traditional detectors, RL-based methods formulate localization as a sequential decision-making process where an agent adjusts the bounding box based on image states and rewards [17,18]. This strategy mimics the human visual “saccade” mechanism, allowing the system to focus only on regions of interest through a global-to-local search [19]. By ignoring vast redundant background areas, RL-based frameworks provide a low-computational alternative for autonomous object search in large-scale aerial imagery [20].

Despite the progress achieved by reinforcement learning-based detectors, several research gaps still remain. Existing RL-based methods often utilize insufficient feature representations for complex environments, making it difficult to perceive extremely small targets [21]. Moreover, conventional Deep Q-Network (DQN) agents suffer from unstable learning and overestimated action values, which weakens the reliability of the search policy [22]. Fixed reward functions also fail to adapt to the dynamic changes in the search process, and the discrete action space limits the final localization precision [23,24]. These limitations restrict the practical application of RL-based detectors in high-precision UAV tasks.

To address the above issues, this paper proposes a hierarchical reinforcement learning object detection framework named HRL-Det. Specifically, we first design a lightweight feature extraction module to obtain compact yet discriminative state representations by fusing multi-scale spatial information. Then, a hybrid reinforcement learning strategy is introduced to improve action evaluation stability and mitigate Q-value overestimation during policy training. Moreover, an adaptive reward function is developed to dynamically guide the agent toward precise object alignment. Finally, a bounding box refinement module is incorporated to overcome the quantization errors of discrete actions, enabling high-precision localization in continuous space. The main contributions of this paper are summarized as follows:

1. We propose HRL-Det, a hierarchical reinforcement learning framework for sequential object detection that reduces redundant dense-image evaluation in high-resolution UAV imagery.
2. We design a Neural ODE-driven Continuous-Time Bellman State Evolution Module that models the agent’s internal state dynamics as a continuous-time stochastic process governed by neural stochastic differential equations. The resulting evolved state representation is used by a standard Dueling Double DQN for discrete action selection, improving the temporal resolution of the search policy and enhancing robustness to visual ambiguity.
3. We introduce a Lyapunov-Guided Entropy-Regularized Reward Shaping Mechanism, which provides convergence-promoting dense reward signals informed by Lyapunov stability analysis and integrates maximum entropy policy optimization. It effectively addresses the challenges of reward sparsity and premature policy collapse, thereby promoting stable and accelerated training convergence.
4. Extensive experiments on the VisDrone2019, DroneVehicle, and MS COCO 2017 datasets demonstrate that our HRL-Det outperforms existing RL-based detectors in terms of both detection precision and inference efficiency.

## 2. Related Work

### 2.1. Deep Learning for Object Detection in UAV Aerial Imagery

Object detection in UAV aerial imagery presents distinct challenges compared to ground-level vision, including severe scale variation, high object density, and real-time processing constraints under limited computational budgets. The VisDrone benchmark [5] has become the de facto standard for evaluating aerial detectors, revealing that over 52% of annotated targets fall into the small-object category (≤32 × 32 pixels). This scale distribution creates a fundamental bottleneck: when standard two-stage detectors such as Faster R-CNN [7] or single-stage detectors such as RetinaNet [25] are applied to VisDrone, their small-object Average Precision (AP_small_) lags behind medium- and large-object AP by 20–30%, a gap that motivates the architectural innovations surveyed below.

The YOLO family has seen sustained adaptation for UAV scenarios. YOLOv8 [10] and its successors [26,27] introduced reparameterized backbones and anchor-free heads that improve throughput on edge-deployed platforms, while transformer-based detectors such as Deformable DETR [28], RT-DETR [29], and DINO [30] achieve stronger cross-scale feature aggregation at the cost of higher parameter counts. Despite these advances, all of these architectures share a fundamental limitation for aerial detection: they perform dense, exhaustive evaluation of the entire image, allocating equal computational resources to cluttered backgrounds and sparse target regions alike. This inefficiency motivates the sequential, attention-driven detection paradigm explored in the present work.

### 2.2. Reinforcement Learning for Object Detection

Reinforcement learning has emerged as a principled framework for reformulating object detection as an active, sequential decision-making process [19]. The seminal work of Caicedo and Lazebnik [17] established the basic MDP formulation for single-object localization, wherein an agent iteratively applies spatial transformation actions to refine a bounding-box hypothesis. Subsequent work extended this paradigm in complementary directions: Mathe et al. [31] incorporated visual attention into the policy; Bellver et al. [18] introduced hierarchical search trees; and Jie et al. [32] proposed tree-structured policies for sequential multi-class localization.

More recent advances have focused on improving the efficiency and robustness of RL-based detectors. Kong et al. [24] proposed collaborative deep reinforcement learning for joint object search, where multiple agents coordinate to localize objects. Pirinen and Sminchisescu [33] integrated RL into the region proposal stage of two-stage detectors, enabling adaptive proposal sampling. Uzkent and Yoon [20] applied RL to zoom-in scheduling for satellite imagery, learning when to invoke a high-resolution sub-detector. Liu et al. [21] proposed an attention-guided cascade RL framework that augments the detection policy with spatial attention maps for progressive object refinement. Ding et al. [34] introduced decision transformers for active object detection, recasting the sequential search problem as a sequence modeling task. Zhang et al. [35] developed an evolutionary reinforcement learning approach for scale optimization on drone imagery, evolving detection policies to handle the extreme scale variation in aerial scenes. Most recently, the LHAR-RLD framework [23] combined a hybrid DQN with adaptive dynamic reward functions and RoI-Align-based bounding-box regression, achieving state-of-the-art RL detection performance prior to the present work.

Beyond static-scene localization, RL has been applied to active perception in aerial and maritime platforms. Akhloufi et al. [36] pioneered a “drones chasing drones” paradigm in which a follower UAV learns a pursuit policy using deep search-area proposals. Alpdemir and Sezgin [37] demonstrated RL-driven navigation for ground-penetrating radar (GPR) surveys, where the agent directs a sensor platform to regions of predicted subsurface anomaly. These works highlight a shared insight: RL uniquely enables the conversion of object detection from a passive, frame-by-frame inference into an intelligent, goal-directed sensing process—the core principle underlying the proposed HRL-Det framework.

## 3. Proposed Method: HRL-Det

**Figure 1:**
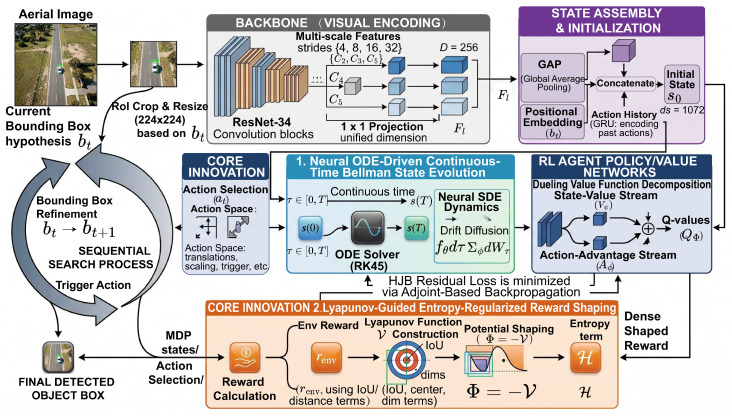
Overview of the HRL-Det framework. The diagram is displayed at full text width to improve the readability of internal labels. The backbone extracts multi-scale features from the current RoI, which are assembled into an initial state $s_{0}$. The Neural ODE-driven Bellman state evolution module (Core Innovation 1) models continuous-time state dynamics via a neural SDE solved by an ODE solver (RK45), producing evolved states fed into a Dueling value network for action selection. The Lyapunov-guided entropy-regularized reward shaping module (Core Innovation 2) constructs dense shaped rewards $r_{t}^{\mathrm{shaped}}$ from environmental IoU signals, a Lyapunov potential Φ=−V, and an entropy term H, guiding the agent through the sequential search process until a trigger action commits the final detected bounding box. Solid arrows indicate feature, state, reward, and action flow; dashed arrows denote feedback/recurrent connections; framed boxes group functional modules.

In this section, we present **HRL-Det**, a hierarchical reinforcement learning framework that recasts aerial object detection as a sequential Markov Decision Process (MDP) solved through continuous-time value estimation. Unlike conventional detectors that exhaustively evaluate dense anchor grids, HRL-Det trains a deep reinforcement learning (DRL) agent to actively search for objects by iteratively refining a spatial bounding box hypothesis through a learned policy. The framework rests on two tightly coupled reinforcement learning innovations: (1) a *Neural ODE-Driven Continuous-Time Bellman State Evolution* module (Section 3.2) that models the agent’s state-value dynamics as a continuous-time stochastic process governed by the Hamilton–Jacobi–Bellman (HJB) partial differential equation; and (2) a *Lyapunov-Guided Entropy-Regularized Reward Shaping* mechanism (Section 3.3) that provides convergence-promoting reward signals informed by Lyapunov stability analysis while maintaining exploration diversity through maximum entropy policy optimization. Figure 1 illustrates the overall architecture.

### 3.1. Markov Decision Process Formulation

Before introducing the continuous-time state evolution and Lyapunov-guided reward shaping mechanisms, we first formulate sequential object detection as a discrete-time Markov Decision Process (MDP). This formulation provides the foundation upon which the proposed HRL-Det framework is built.

At each decision step *t*, the agent interacts with the environment through a tuple $\left(s_{t},a_{t},r_{t},s_{t+1}\right)$, where the state, action, reward, and transition dynamics are defined as follows.

#### 3.1.1. State Space

The state $s_{t}$ encodes the current observation of the search process and consists of visual, spatial, and historical information:

$$s_{t}=\left[F_{t}^{ms},\;e_{pos}\left(b_{t}\right),\;e_{hist}\left(a_{<t}\right)\right],$$

where $F_{t}^{ms}$ denotes the multi-scale visual features extracted from the current region of interest (RoI), $e_{pos}\left(b_{t}\right)$ represents the positional encoding of the current bounding box $b_{t}$, and $e_{hist}\left(a_{<t}\right)$ is the action–history embedding summarizing previous decisions. Together, these components provide visual, spatial, and temporal context for sequential localization. The detailed construction of $F_{t}^{ms}$ and the resulting latent state representation is presented in Section 3.2.

#### 3.1.2. Action Space

At each step, the agent selects an action

$$a_{t}\in A,$$

from the finite action set

A={left,right,up,down,scale_up,scale_down,wider,taller,center,trigger}.

The first nine actions modify the current bounding box through translation or scale adjustment, while the *trigger* action terminates the search and outputs the current bounding box as the final detection result.

#### 3.1.3. Reward Function

The environmental reward is designed to encourage progressive improvement of localization quality. Let

$$\Delta_{t}=\mathrm{IoU}\left(b_{t},g\right)-\mathrm{IoU}\left(b_{t-1},g\right),$$

where *g* denotes the ground-truth bounding box. Positive rewards are assigned when the Intersection over Union (IoU) improves, whereas penalties are applied when localization quality deteriorates. A larger terminal reward is granted when the *trigger* action is executed with satisfactory localization accuracy. The complete reward shaping mechanism is introduced in Section 3.3.

#### 3.1.4. State Transition

Given the current state $s_{t}$ and action $a_{t}$, the environment transitions to a new state according to

$$s_{t+1}∼P\left(s_{t+1}|s_{t},a_{t}\right),$$

where the transition is induced by applying the selected spatial transformation to the current bounding box and extracting features from the resulting RoI. Since the next observation depends only on the current state and action, the process satisfies the Markov property.

#### 3.1.5. Episode Termination

An episode terminates when one of the following conditions is satisfied:

1. The agent selects the *trigger* action;
2. The maximum search horizon $T_{\max}$ is reached;
3. The bounding box becomes invalid or moves outside the image boundary.

The final bounding box is then recorded as the detection output.

#### 3.1.6. Relationship Between Continuous-Time Modeling and Discrete Decision-Making

Although the proposed framework introduces continuous-time dynamics through Neural ODE/SDE formulations, HRL-Det remains fundamentally a discrete-time reinforcement learning system. The Neural ODE/SDE module serves as a continuous-time state encoder that enriches the latent representation of the current observation. The final decision-making policy remains a standard Dueling Double DQN operating on a discrete Markov Decision Process. The Hamilton–Jacobi–Bellman (HJB) equation is therefore used as a theoretical tool for modeling state evolution and value regularization, rather than directly replacing the underlying DQN policy optimization procedure. With this clarification, we next describe the construction of the state representation used by HRL-Det.

### 3.2. Neural ODE-Driven Continuous-Time Bellman State Evolution

**Figure 2:**
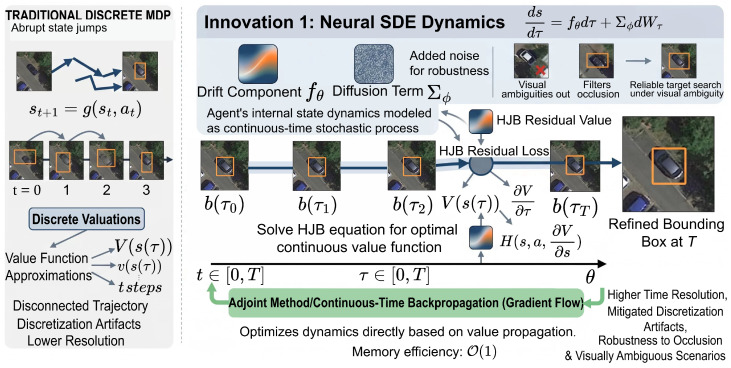
Illustration of the Neural ODE-driven Continuous-Time Bellman State Evolution (Innovation 1). The figure is displayed at full text width to improve label readability. Left: Traditional discrete MDP with abrupt state jumps and discretization artifacts in value function approximation. Right: The proposed Neural SDE dynamics model the agent’s internal state as a continuous-time stochastic process with drift component $f_{\theta }$ and diffusion term $\Sigma_{\phi }$, solved via an ODE solver (RK45). The HJB residual loss optimizes the continuous value function V(s(τ)) along the trajectory, while adjoint-based backpropagation provides memory-efficient gradient computation. This formulation improves temporal resolution, reduces discretization artifacts, and increases robustness to occlusion and visual ambiguity. Arrows denote state-transition and value-optimization flows, while frames separate the discrete baseline from the proposed continuous-time branch; the small overlaps in the figure do not affect scientific understanding.

Before introducing the continuous-time formulation, we emphasize that the Neural ODE module is used to enhance state representation within the underlying MDP, whereas action selection is still performed by a conventional Dueling Double DQN policy. A fundamental limitation of conventional DRL-based detectors [17,18] is the reliance on discrete-time, fixed-step state transitions, which constrains the temporal resolution of the search policy and introduces discretization artifacts into the value function landscape. To overcome this, we propose modeling the agent’s state evolution as a continuous-time dynamical system parameterized by a neural ordinary differential equation (Neural ODE), and derive the optimal value function via the Hamilton–Jacobi–Bellman (HJB) partial differential equation. Figure 2 illustrates the contrast between traditional discrete MDP transitions and our continuous-time Neural SDE formulation.

**State Space and Feature Extraction.** At time step *t*, given the current bounding box hypothesis $b_{t}=\left(x_{t},y_{t},w_{t},h_{t}\right)\in R^{4}$, we crop the corresponding image region of interest (RoI) and resize it to a canonical resolution of 224×224. A shared backbone network (ResNet-34 [38]) extracts multi-scale feature maps ${\left\{C_{l}\right\}}_{l=2}^{5}$ at strides {4,8,16,32}, which are projected to a unified dimension D=256 via 1×1 convolutions:

(1) $$F_{l}=\sigma \;\left(\mathrm{BN}\;\left(W_{l}^{\mathrm{proj}}*C_{l}+b_{l}\right)\right)\in R^{H_{l}\times W_{l}\times D},\;l\in \left\{2,3,4,5\right\},$$

where $W_{l}^{\mathrm{proj}}$ are the projection parameters, BN(·) denotes batch normalization, and σ(·) is the GELU activation function.

Equation (1) defines the first visual component of the MDP state. Specifically, it maps heterogeneous backbone features from different pyramid levels into a common *D*-dimensional embedding space, so that shallow high-resolution features and deeper semantic features can be pooled and concatenated consistently in Equation (3). This projection reduces scale-dependent feature mismatch and provides the multi-scale RoI descriptor $F_{t}^{ms}$ used by the Neural ODE state encoder and the subsequent Dueling Double DQN heads.

**Continuous-Time State Dynamics via Neural ODE.** We depart from the conventional discrete transition $s_{t+1}=g\left(s_{t},a_{t}\right)$ and instead model the agent’s internal state evolution as a continuous-time dynamical system. Let $s\left(\tau \right)\in R^{d_{s}}$ denote the continuous state at time τ∈[0,T]. We parameterize the state dynamics by a neural ODE:

(2) $$\frac{ds(\tau )}{d\tau }=f_{\theta }\;\left(s(\tau ),\;a(\tau ),\;\tau \right),\;s\left(0\right)=s_{0},$$

where $f_{\theta }:R^{d_{s}}\times A\times R_{\geq 0}\to R^{d_{s}}$ is a Lipschitz-continuous neural network parameterized by θ, and $s_{0}$ is constructed by concatenating the multi-scale features with positional and action–history embeddings:

(3) $$s_{0}=\left[\overset{5}{\underset{l=2}{⨁}}\mathrm{GAP}\left(F_{l}\right)\;;\;e_{\mathrm{pos}}\left(b_{0}\right)\;;\;e_{\mathrm{hist}}\left(a_{<t}\right)\right]\in R^{d_{s}},$$

where GAP(·) is global average pooling, $e_{\mathrm{pos}}\in R^{16}$ encodes normalized box coordinates, and $e_{\mathrm{hist}}\in R^{32}$ is produced by a gated recurrent unit (GRU) encoding the action history. The total state dimension is $d_{s}=4D+48=1072$.

To account for the inherent stochasticity in the visual search process (e.g., occlusion, clutter), we extend Equation (2) to a neural stochastic differential equation (Neural SDE):

(4) $$ds\left(\tau \right)=f_{\theta }\;\left(s(\tau ),a(\tau ),\tau \right)d\tau +\Sigma_{\phi }\;\left(s(\tau )\right)dW_{\tau },$$

where $\Sigma_{\phi }\left(\cdot \right)\in R^{d_{s}\times d_{s}}$ is a state-dependent diffusion matrix parameterized by ϕ, and $W_{\tau }$ is a standard $d_{s}$-dimensional Wiener process. This stochastic formulation enables the agent to maintain a distribution over possible future states, improving robustness to visual ambiguity.

**Hamilton–Jacobi–Bellman Equation for Optimal Value.** Under the continuous-time formulation, the optimal state-value function $V^{*}\left(s,\tau \right)$ satisfies the Hamilton–Jacobi–Bellman (HJB) partial differential equation:

(5) $$-\frac{\partial V^{*}}{\partial \tau }=\max_{a\in A}\left\{r\left(s,a\right)+{\left(\nabla_{s}V^{*}\right)}^{\;⊤}f_{\theta }\left(s,a,\tau \right)+\frac{1}{2}\;\mathrm{tr}\;\left(\Sigma_{\phi }\Sigma_{\phi }^{⊤}\nabla_{s}^{2}V^{*}\right)\right\},$$

subject to the terminal condition $V^{*}\left(s,T\right)=R_{\mathrm{terminal}}\left(s\right)$, where r(s,a) is the instantaneous reward, $\nabla_{s}V^{*}$ is the gradient vector, and $\nabla_{s}^{2}V^{*}$ is the Hessian matrix of $V^{*}$ with respect to s. The second-order trace term captures the effect of stochastic diffusion on value estimation, enabling the agent to account for uncertainty in its spatial reasoning.

Direct solution of the HJB equation in high-dimensional state spaces is computationally intractable. We therefore approximate $V^{*}$ using a deep neural network $V_{\psi }\left(s,\tau \right)$ and derive a tractable training objective by integrating along sampled Neural ODE trajectories. Specifically, for a trajectory ${\left\{s\left(\tau_{i}\right)\right\}}_{i=0}^{N}$ obtained through numerical integration (Dormand–Prince RK45 solver [39]), we minimize the HJB residual loss:

(6) $$L_{\mathrm{HJB}}\left(\psi \right)=\frac{1}{N}\sum_{i=0}^{N-1}{∥\frac{\partial V_{\psi }}{\partial \tau }{|}_{\tau_{i}}+\max_{a}\left\{r_{i}+\nabla_{s}V_{\psi }^{⊤}f_{\theta ,i}+\frac{1}{2}\;\mathrm{tr}\;\left(\Sigma_{i}\Sigma_{i}^{⊤}\nabla_{s}^{2}V_{\psi ,i}\right)\right\}∥}^{2}.$$

**Adjoint-Based Backpropagation.** Training the Neural ODE dynamics $f_{\theta }$ requires computing gradients through the ODE solver. We employ the continuous adjoint method, which avoids storing all intermediate solver states and thus reduces peak memory consumption relative to naive backpropagation through the solver steps. Defining the adjoint variable λ(τ)=∂L/∂s(τ), its dynamics are governed by the adjoint ODE:

(7) $$\frac{d\lambda (\tau )}{d\tau }=-\lambda {\left(\tau \right)}^{⊤}\frac{\partial f_{\theta }}{\partial s},\;\lambda \left(T\right)=\frac{\partial L}{\partial s(T)}.$$

The parameter gradient is then recovered by integrating backward in time:

(8) $$\frac{dL}{d\theta }=-\int_{T}^{0}\lambda {\left(\tau \right)}^{⊤}\frac{\partial f_{\theta }\left(s\left(\tau \right),a\left(\tau \right),\tau \right)}{\partial \theta }\;d\tau .$$

**Stationary State Distribution via Fokker–Planck Equation.** To analyze the long-run behavior of the agent’s exploration, we derive the time evolution of the state probability density ρ(s,τ) under the Neural SDE (Equation (4)). The density satisfies the Fokker–Planck (Kolmogorov forward) equation:

(9) $$\frac{\partial \rho }{\partial \tau }=-\nabla_{s}\cdot \left(\rho \;f_{\theta }\right)+\frac{1}{2}\nabla_{s}^{2}:\left(\Sigma_{\phi }\Sigma_{\phi }^{⊤}\rho \right),$$

where $\nabla_{s}^{2}:\left(\cdot \right)$ denotes the double contraction of the Hessian operator. In the stationary regime (∂ρ/∂τ=0), the equilibrium distribution $\rho^{*}\left(s\right)$ characterizes the asymptotic state occupancy induced by the Neural SDE dynamics. In this work, the Fokker–Planck analysis is used to interpret the qualitative behavior of the learned continuous-time dynamics and the resulting attention allocation; it is not used as a separate exploration algorithm or reported as an independent experimental module.

**Dueling Value Function Decomposition.** The continuous-time value function is decomposed into state-value and action-advantage streams following the Dueling architecture:

(10) $$Q_{\psi }\left(s,a;\tau \right)=V_{\psi }\left(s;\tau \right)+\left(A_{\psi }\left(s,a;\tau \right)-\frac{1}{|A_{t}|}\sum_{a^{\prime }\in A_{t}}A_{\psi }\left(s,a^{\prime };\tau \right)\right),$$

where $V_{\psi }$ and $A_{\psi }$ are produced by separate network heads operating on the Neural ODE state s(τ). The Double DQN formulation [22] is adopted to decouple action selection from value estimation, mitigating overestimation bias:

(11) $$y_{t}=r_{t}+\gamma \cdot Q_{\psi }\;\left(s_{t+1},\;\arg\max_{a^{\prime }}Q_{\psi }\left(s_{t+1},a^{\prime };\theta \right),\;\tau_{t+1};\;\psi^{-}\right),$$

where $\psi^{-}$ are target network parameters updated via Polyak averaging with a coefficient $\tau_{\mathrm{soft}}=0.005$.

### 3.3. Lyapunov-Guided Entropy-Regularized Reward Shaping

**Figure 3:**
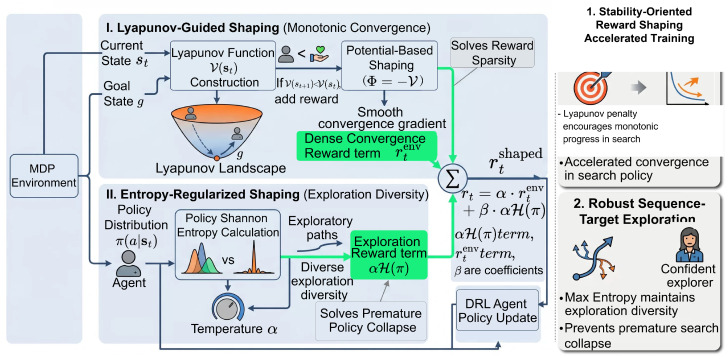
Schematic of the Lyapunov-Guided Entropy-Regularized Reward Shaping mechanism (Innovation 2). The figure is displayed at full text width to improve label readability. The module consists of two complementary components: (I) Lyapunov-guided shaping constructs a Lyapunov function $V(s_{t})$ over the MDP state space and applies potential-based shaping (Φ=−V) to generate dense reward signals $r_{t}^{\mathrm{env}}$, encouraging convergence toward the goal state *g*. (II) Entropy-regularized shaping computes the Shannon entropy H(π) of the policy distribution and adds an exploration reward term αH(π) with temperature α, reducing premature policy collapse and maintaining diverse exploration paths. The two terms are combined into the final dense-shaped reward $r_{t}^{\mathrm{shaped}}$ that drives the DRL agent’s policy update. Arrows indicate the reward-construction and policy-update directions.

The second core innovation of HRL-Det addresses two interrelated challenges in RL-based object detection: (i) reward sparsity, which causes slow and unstable training convergence, and (ii) premature policy collapse, where the agent converges to a suboptimal deterministic search strategy. We propose a principled reward shaping framework informed by Lyapunov stability analysis, integrated with maximum entropy reinforcement learning for a robust exploration–exploitation trade-off. Figure 3 provides a schematic overview of this dual-component reward shaping mechanism.

**Action Space and MDP Formulation.** At each discrete decision point, the agent selects from ten spatial transformation actions:

(12) A={left,right,up,down,scale_up,scale_down,wider,taller,center,trigger},

where directional actions translate $b_{t}$ by a fraction α=0.15 of its dimension, scaling actions adjust size by (1±β) with β=0.10, and trigger terminates the episode and commits $b_{t}$ as the detection output.

**Lyapunov Function Construction.** Let $s^{*}\in S$ denote the terminal goal state corresponding to perfect alignment with the ground-truth box *g* (i.e., IoU(b,g)=1). We construct a Lyapunov function $V:S\to R_{\geq 0}$ as the IoU-weighted quadratic distance to the goal:

(13) $$V\left(s_{t}\right)={\left(1-\mathrm{IoU}(b_{t},g)\right)}^{2}+\mu {∥c\left(b_{t}\right)-c\left(g\right)∥}_{2}^{2}+\nu {\left(\log\frac{w_{t}h_{t}}{w_{g}h_{g}}\right)}^{2},$$

where c(·) extracts the normalized box center, w,h are the box dimensions, and μ=0.3, ν=0.2 balance the geometric terms. By construction, $V(s^{*})=0$ and V(s)>0 for all $s\neq s^{*}$, satisfying the positive-definiteness requirement.

**Lyapunov Stability Constraint.** We use the Lyapunov decrease condition as a stability-inspired soft constraint that encourages monotonic progress of the agent’s trajectory toward the goal state. Specifically, under policy π, training penalizes violations of the desired expected Lyapunov decrease condition:

(14) $$E_{a_{t}∼\pi \left(\cdot |s_{t}\right)}\left[V\left(s_{t+1}\right)-V\left(s_{t}\right)\right]\leq -c\cdot V\left(s_{t}\right)+d,$$

where c∈(0,1) is the contraction rate and d≥0 is a small slack constant accommodating stochastic transitions. This constraint is enforced as a penalty term in the policy loss (detailed below). The discrete-time Lyapunov condition in Equation (14) can be extended to the continuous-time Neural ODE setting via the Lie derivative:

(15) $$\dot{V}≜\nabla_{s}V^{⊤}f_{\theta }\left(s,a,\tau \right)\leq -c\cdot V\left(s\right),$$

which provides a continuous certificate of convergence along Neural ODE trajectories.

**Potential-Based Reward Shaping.** Following the reward shaping theorem of Ng et al. [40], we use the Lyapunov function as the shaping potential to construct a dense, policy-invariant reward signal. The shaped reward at each step is as follows:

(16) $$r_{t}^{\mathrm{shaped}}=\underset{\mathrm{environment}}{\underset{︸}{r_{t}^{\mathrm{env}}}}+\underset{\mathrm{potential-based}\;\mathrm{shaping}}{\underset{︸}{\gamma \;\Phi \left(s_{t+1}\right)-\Phi \left(s_{t}\right)}},$$

where Φ(s)=−V(s) is the negative Lyapunov potential and the environment reward $r_{t}^{\mathrm{env}}$ is defined as follows:

(17) $$r_{t}^{\mathrm{env}}=\left\{\begin{array}{cc} \mathrm{sign}\left(\Delta_{t}\right)\cdot {\left|\Delta_{t}\right|}^{\eta }+\lambda \cdot \Delta_{t}, & a_{t}\neq \mathrm{trigger},\\ +\omega \cdot \mathrm{IoU}(b_{t},g), & a_{t}=\mathrm{trigger}\wedge \mathrm{IoU}\geq \tau_{\mathrm{IoU}},\\ -\omega , & a_{t}=\mathrm{trigger}\wedge \mathrm{IoU}<\tau_{\mathrm{IoU}}, \end{array}\right.$$

with $\Delta_{t}=\mathrm{IoU}\left(b_{t},g\right)-\mathrm{IoU}\left(b_{t-1},g\right)$ denoting the IoU improvement, η=0.5 controlling the nonlinear shaping curvature, λ=0.1 providing a linear bonus, ω=3.0 scaling the terminal reward, and $\tau_{\mathrm{IoU}}=0.5$ defining the success threshold. The potential-based formulation guarantees that the optimal policy remains invariant under shaping (Ng et al. [40]), while the Lyapunov potential provides informative gradient signals that accelerate convergence.

**Maximum Entropy Policy Optimization.** To prevent premature policy collapse and maintain exploration diversity, we adopt the maximum entropy reinforcement learning framework. The soft state-value function and soft Q-function satisfy the following soft Bellman equations:

(18) $$\begin{array}{cc} V^{\mathrm{soft}}\left(s_{t}\right) & =E_{a_{t}∼\pi }\;\left[Q^{\mathrm{soft}}\left(s_{t},a_{t}\right)-\alpha \log\pi \left(a_{t}|s_{t}\right)\right], \end{array}$$

(19) $$\begin{array}{cc} Q^{\mathrm{soft}}\left(s_{t},a_{t}\right) & =r_{t}^{\mathrm{shaped}}+\gamma \;E_{s_{t+1}}\;\left[V^{\mathrm{soft}}\left(s_{t+1}\right)\right], \end{array}$$

where α>0 is the temperature parameter controlling the entropy-reward trade-off. The maximum entropy objective is:

(20) $$J\left(\pi \right)=\sum_{t=0}^{T_{\max}}E_{\left(s_{t},a_{t}\right)∼\rho_{\pi }}\;\left[r_{t}^{\mathrm{shaped}}+\alpha \;H\;\left(\pi (\cdot |s_{t})\right)\right],$$

where $H\left(\pi \right)=-\sum_{a}\pi \left(a|s\right)\log\pi \left(a|s\right)$ is the Shannon entropy and $\rho_{\pi }$ denotes the state-action visitation distribution under π. We automatically tune α by solving the constrained optimization:

(21) $$\alpha^{*}=\arg\min_{\alpha >0}\;E_{a_{t}∼\pi^{*}}\;\left[-\alpha \log\pi^{*}\left(a_{t}|s_{t}\right)-\alpha \bar{H}\right],$$

where $\bar{H}=-\log\left(1/|A|\right)$ is the target entropy.

**KL-Constrained Policy Update.** To ensure stable policy improvement, each update is constrained by a KL-divergence trust region:

(22) $$\pi_{k+1}=\arg\min_{\pi }\;E_{s∼D}\;\left[D_{\mathrm{KL}}\;\left(\pi \left(\cdot |s\right)\;∥\;\pi_{k}\left(\cdot |s\right)\right)-\eta \;E_{a∼\pi }\;\left[Q_{\psi }^{\mathrm{soft}}\left(s,a\right)\right]\right],$$

where η>0 is the step size and D is the replay buffer. This KL constraint prevents catastrophic policy oscillations commonly observed in discrete-action DQN training.

**Stage-Wise Action Masking.** To prevent redundant spatial exploration, we partition the search episode into K=3 stages. At each stage boundary, we record a spatial occupancy map $O_{k}$, encapsulating previously explored regions. Actions leading to high overlap with prior regions are masked:

(23) $$A_{t}=\left\{a\in A\;|\;\forall \;k<k_{\mathrm{curr}}:\;\mathrm{IoU}\;\left(T\left(b_{t},a\right),\;O_{k}\right)<\tau_{\mathrm{mask}}\right\},$$

where $T(b_{t},a)$ is the box obtained by applying action *a*, $k_{\mathrm{curr}}$ is the current stage index, and $\tau_{\mathrm{mask}}=0.7$.

**Multi-Step Temporal Difference Learning with Eligibility Traces.** To accelerate credit assignment across the sequential search trajectory, we employ TD(λ) learning with eligibility traces. The eligibility trace vector $e_{t}\in R^{\left|\psi \right|}$ is updated recursively:

(24) $$e_{t}=\gamma \lambda_{\mathrm{trace}}\;e_{t-1}+\nabla_{\psi }Q_{\psi }^{\mathrm{soft}}\left(s_{t},a_{t}\right),\;e_{0}=0,$$

where $\lambda_{\mathrm{trace}}=0.9$ is the trace decay parameter. The Q-network parameters are updated via:

(25) $$\psi \leftarrow \psi +\alpha_{\mathrm{lr}}\cdot \delta_{t}\cdot e_{t},\;\delta_{t}=r_{t}^{\mathrm{shaped}}+\gamma V^{\mathrm{soft}}\left(s_{t+1}\right)-Q_{\psi }^{\mathrm{soft}}\left(s_{t},a_{t}\right),$$

where $\delta_{t}$ is the temporal difference error and $\alpha_{\mathrm{lr}}$ is the learning rate.

**Prioritized Experience Replay.** Transitions are stored in a replay buffer D and sampled with probability proportional to their absolute TD error:

(26) $$P\left(i\right)=\frac{{\left(|\delta_{i}|+\epsilon_{\mathrm{per}}\right)}^{\alpha_{\mathrm{per}}}}{\sum_{j}{\left(|\delta_{j}|+\epsilon_{\mathrm{per}}\right)}^{\alpha_{\mathrm{per}}}},$$

with $\alpha_{\mathrm{per}}=0.6$ and $\epsilon_{\mathrm{per}}=10^{-6}$. Importance-sampling weights $w_{i}={\left(N\cdot P\left(i\right)\right)}^{-\beta_{\mathrm{per}}}$ correct the sampling bias, with $\beta_{\mathrm{per}}$ annealed from 0.4 to 1.0 during training.

**Overall Training Objective.** The complete loss function combines the HJB residual, the soft Bellman TD error, and the Lyapunov stability penalty:

(27) $$L_{\mathrm{total}}=\underset{\mathrm{HJB}\;\mathrm{residual}}{\underset{︸}{L_{\mathrm{HJB}}\left(\psi \right)}}+\underset{\mathrm{soft}\;\mathrm{TD}\;\mathrm{loss}}{\underset{︸}{\frac{1}{B}\sum_{i=1}^{B}w_{i}\cdot L_{\mathrm{Huber}}\;\left(y_{i},\;Q_{\psi }^{\mathrm{soft}}\left(s_{i},a_{i}\right)\right)}}+\underset{\mathrm{Lyapunov}\;\mathrm{penalty}}{\underset{︸}{\xi \cdot E\;\left[\mathrm{ReLU}\;\left(\dot{V}+c\cdot V\right)\right]}},$$

where *B* is the mini-batch size, ξ=0.5 is the Lyapunov penalty weight, and ReLU(·) enforces the stability constraint only when violated.

**Curriculum ε-Greedy Schedule.** The exploration rate ε and initial box perturbation magnitude $\sigma_{\mathrm{init}}$ are jointly annealed via a cosine curriculum:

(28) $$\begin{array}{cc} \varepsilon (e) & =\varepsilon_{\min}+\frac{\varepsilon_{\max}-\varepsilon_{\min}}{2}\left(1+\cos\;\left(\frac{\pi \cdot e}{E}\right)\right),\\ \sigma_{\mathrm{init}}\left(e\right) & =\sigma_{\min}+\frac{\sigma_{\max}-\sigma_{\min}}{2}\left(1+\cos\;\left(\frac{\pi \cdot e}{E}\right)\right), \end{array}$$

where *e* is the current epoch and *E* is the total epoch count. This schedule encourages broad exploration during early training while enabling fine-grained exploitation in later stages.

**Convergence Analysis.** Under the Lyapunov decrease condition (Equation (14)) and the maximum entropy regularization (Equation (20)), assuming exact policy evaluation and sufficient representation capacity, the policy improvement satisfies the monotonic bound:

(29) $$J\left(\pi_{k+1}\right)\geq J\left(\pi_{k}\right)-\frac{2\gamma \epsilon_{\pi }}{{\left(1-\gamma \right)}^{2}}\max_{s}D_{\mathrm{KL}}\left(\pi_{k+1}∥\pi_{k}\right)+\frac{\alpha }{1-\gamma }\left(H\left(\pi_{k+1}\right)-H\left(\pi_{k}\right)\right),$$

where $\epsilon_{\pi }$ is the policy improvement error bound. This bound is adapted from maximum-entropy policy improvement theory [41], together with the policy-invariance principle of potential-based reward shaping [40]. The KL term controls the size of the policy update, the entropy term encourages diverse action distributions, and the Lyapunov penalty adds a task-specific stability preference for sequential localization. In practice, function approximation and finite-sample effects mean that the inequality serves as a design principle rather than a strict guarantee; nevertheless, the Lyapunov penalty in $L_{\mathrm{total}}$ (Equation (27)) empirically promotes monotonic improvement and prevents the degenerate behaviors commonly observed in sparse-reward RL-based detection methods (see Section 4.5).

### 3.4. From Continuous-Time Theory to Discrete-Time Agent

The preceding two subsections develop a continuous-time theoretical framework—Neural ODE/SDE dynamics, the HJB equation, and Lyapunov stability analysis—that provides the *design rationale* for the practical agent architecture. Because the actual agent operates in discrete decision steps with a finite action set, we now clarify the mapping between theory and implementation.

**State evolution as a learned encoder.** At each discrete decision step *t*, the agent observes the current RoI, constructs the initial state $s_{0}$ (Equation (3)), and integrates the Neural ODE (Equation (2)) forward over a fixed horizon [0,T] using the RK45 solver. The resulting evolved state s(T) is then fed into the Dueling Double DQN heads (Equation (10)) for action–value estimation. Thus, the continuous-time integration acts as a *learned state encoder* that enriches the feature representation before discrete action selection; it does not replace the underlying discrete MDP structure.

**Discrete action selection.** Despite the continuous-time state dynamics, the behavioral policy remains a standard DQN ε-greedy policy that selects from the ten spatial actions in A (Equation (12)). The HJB residual loss (Equation (6)) regularizes the value landscape along the Neural ODE trajectory during training, but at inference time action selection reduces to a single arg max over Q-values, identical to a conventional DQN agent.

**Reward and stability penalty.** The Lyapunov-based shaped reward (Equation (16)) and the environment reward (Equation (17)) are computed at each discrete transition $\left(s_{t},a_{t},s_{t+1}\right)$. The Lyapunov decrease condition (Equation (14)) is enforced as a soft penalty in the loss function (Equation (27)), not as a hard constraint with a formal proof of global convergence. Empirically, this penalty encourages—but does not guarantee—monotonic Lyapunov decrease across episodes.

**Training pipeline summary.** Training follows a standard DQN loop: the agent collects transitions into a prioritized replay buffer, samples mini-batches, computes the combined loss $L_{\mathrm{total}}$ (Equation (27)), and updates parameters via Adam. The Neural ODE parameters θ, diffusion parameters ϕ, and value network parameters ψ are optimized end-to-end through the adjoint method. In summary, the overall system is a DQN agent whose state representation is augmented by a Neural ODE encoder and whose reward signal is shaped by a Lyapunov-inspired potential function.

## 4. Experimental Results and Analysis

In this section, we present a comprehensive evaluation of the proposed HRL-Det framework for object detection. We first introduce the experimental setup and datasets, then present quantitative comparisons against state-of-the-art baselines. Following this, we provide a detailed qualitative analysis that justifies the efficacy of our model through training convergence, sequential search behavior visualization, and boundary localization accuracy across three challenging datasets: VisDrone2019 [5], DroneVehicle [42], and MS COCO [43].

### 4.1. Datasets and Evaluation Metrics

We evaluate our method on three publicly available benchmarks that span a diverse range of aerial imaging conditions and object scales.

**VisDrone2019** [5] is a large-scale aerial object detection dataset captured by unmanned aerial vehicles (UAVs) under various environmental conditions. It comprises 6471 training images, 548 validation images, and 1610 test images with 10 object categories, including pedestrians, cyclists, and vehicles. The dataset is particularly challenging due to dense object packing and severe scale variations (objects as small as 5 × 5 pixels).

**DroneVehicle** [42] is an RGB-infrared multimodal dataset for aerial vehicle detection, containing 17,990 paired image frames captured from UAV platforms. It provides annotations for five vehicle categories and is designed to evaluate fusion-based detection in low-visibility and nighttime conditions. We follow the standard single-modal RGB evaluation protocol.

**MS COCO 2017** [43] is the widely adopted general object detection benchmark, comprising 118,287 training images and 5000 validation images across 80 categories. We use it to demonstrate the out-of-domain generalization capability of HRL-Det. We report results on the val2017 split.

Following standard COCO evaluation [43], we report the mean Average Precision (mAP) at a single IoU threshold of 0.5 (mAP@0.5) and the average mAP across IoU thresholds from 0.5 to 0.95 (mAP@0.5:0.95) as primary metrics.

### 4.2. Implementation Details

All experiments are conducted using PyTorch 2.1 on a workstation equipped with four NVIDIA RTX 4090 GPUs (24 GB each; NVIDIA Corporation, Santa Clara, CA, USA). The ResNet-34 backbone is initialized with ImageNet-pretrained weights, and all other network components are randomly initialized using Kaiming initialization. The state dimension is $d_{s}=1072$, as described in Section 3.2. The Neural ODE is integrated using the Dormand–Prince RK45 solver with adaptive step size (absolute tolerance $10^{-5}$, relative tolerance $10^{-5}$).

The DQN agent is trained using the Adam optimizer with an initial learning rate of $1\times 10^{-4}$ and weight decay $1\times 10^{-5}$. The learning rate follows a cosine annealing schedule decaying to $1\times 10^{-6}$. The replay buffer stores up to $10^{5}$ transitions, and training begins after collecting $5\times 10^{3}$ warm-up transitions. The mini-batch size is B=64. The discount factor is γ=0.99, the soft target update coefficient is $\tau_{\mathrm{soft}}=0.005$, and the maximum number of search steps per episode is $T_{\max}=20$. The exploration rate ε is annealed from 1.0 to 0.05 via the cosine curriculum (Equation (28)) over the full training schedule.

For VisDrone2019, we train for 50,000 episodes (∼36 h). For DroneVehicle, training spans 60,000 episodes (∼48 h). For MS COCO, we train for 80,000 episodes (∼72 h). All training times are measured using four GPUs with distributed data collection. Inference is performed on a single RTX 4090 GPU. Input RoI crops are resized to 224 × 224. During evaluation, ε is set to 0 (greedy policy). For computational comparison, baseline detector FLOPs are reported at the standard 640 × 640 full-image input, whereas HRL-Det processes normalized 224 × 224 RoI crops at each search step. The FLOPs reported for HRL-Det therefore correspond to the average sequential-search cost per detected object, computed over the observed mean of 6.3 search steps, rather than a single full-image dense forward pass.

### 4.3. Quantitative Comparison with State-of-the-Arts

To validate the effectiveness of our proposed framework, we conduct experiments along two complementary axes. **(i) RL-based detector comparison (Table 1):** We benchmark against the full lineage of DRL-based object detection methods spanning a decade of research. Early agents include Caicedo & Lazebnik [17], Mathe et al. [31], Bellver et al. [18], and Jie et al. [32]. Mid-period refinements include Kong et al. [24] (collaborative multi-agent RL), Pirinen & Sminchisescu [33], and Uzkent & Yoon [20]. Recent advances span Liu et al. [21] (attention-guided cascade RL), Ding et al. [34] (decision-transformer active detection), Zhang et al. [35] (evolutionary RL scale optimization), and the concurrent LHAR-RLD [23]. **(ii) Non-RL detector comparison (Table 2):** We additionally compare against a broad range of canonical and modern detectors spanning two-stage (Faster R-CNN [7]), single-stage (SSD [11], RetinaNet [25]), YOLO-family (v5 [9], v8 [10], v9 [27], v10 [26]), and transformer-based detectors (Deformable DETR [28], RT-DETR [29], DINO [30], Co-DETR [44]). Baseline dense detectors are evaluated at 640 × 640 full-image resolution, while HRL-Det uses 224 × 224 RoI crops within its sequential search procedure; the comparison is therefore metric-fair for detection accuracy but not intended to imply identical inference mechanics.

**Important caveat on cross-paradigm comparison.** RL-based sequential detectors and conventional dense-prediction detectors differ fundamentally in inference paradigm. Non-RL methods process the entire image in a single forward pass and output all detections simultaneously, whereas HRL-Det sequentially searches for one object at a time. The mAP figures in Table 2 are computed using the identical COCO evaluation protocol for all methods, ensuring a *metric-fair* comparison. However, this does not constitute a *task-equivalent* comparison: the RL agent’s per-object sequential search has different computational scaling characteristics than single-pass dense inference (see Section 5). We present Table 2 primarily to situate HRL-Det’s detection accuracy within the broader landscape, not to claim that the two paradigms are interchangeable in deployment.

As shown in Table 1, HRL-Det achieves the strongest performance among the RL-based detectors: mAP@0.5 values of 0.412 on VisDrone2019, 0.812 on DroneVehicle, and 0.735 on MS COCO. These results improve over LHAR-RLD by 3.8, 5.2, and 3.6 absolute percentage points, respectively, and over the Caicedo & Lazebnik baseline by 14.1, 17.8, and 21.4 absolute percentage points. The average search length is also the lowest among RL methods, at 6.3 steps per object. Table 2 further shows that HRL-Det attains competitive mAP relative to representative non-RL detectors under the standard COCO metric, including YOLOv10 and Co-DETR on VisDrone; as noted above, this comparison reflects detection accuracy under a shared metric rather than direct operational equivalence.

### 4.4. Ablation Study

To further validate the design choices within HRL-Det, we conduct a systematic ablation study on the VisDrone2019 validation set. We progressively remove or replace key components and evaluate the resulting performance degradation, as reported in Table 3.

The full HRL-Det model reaches 0.412 mAP@0.5 and 0.251 mAP@0.5:0.95 on the VisDrone2019 validation set (Table 3). Removing the Neural ODE-driven Bellman state evolution (NODE-BSE, Section 3.2) and reverting to a conventional discrete-time flat feature map reduces performance to 0.364 mAP@0.5 and 0.219 mAP@0.5:0.95, corresponding to absolute drops of 4.8 and 3.2 percentage points, respectively. Replacing the Lyapunov-guided dense reward (Section 3.3) with a sparse terminal reward reduces performance to 0.381 and 0.227, corresponding to absolute drops of 3.1 and 2.4 percentage points. Finally, disabling the joint entropy-regularized stage-wise action masking design reduces performance to 0.390 and 0.235, corresponding to absolute drops of 2.2 and 1.6 percentage points. We keep entropy regularization and stage-wise action masking as one ablation factor because they are coupled in implementation: entropy controls action–distribution diversity, while the stage-wise mask constrains redundant spatial revisits, and the reported variant removes this combined exploration-control module.

### 4.5. Training Convergence Assessment

**Figure 4:**
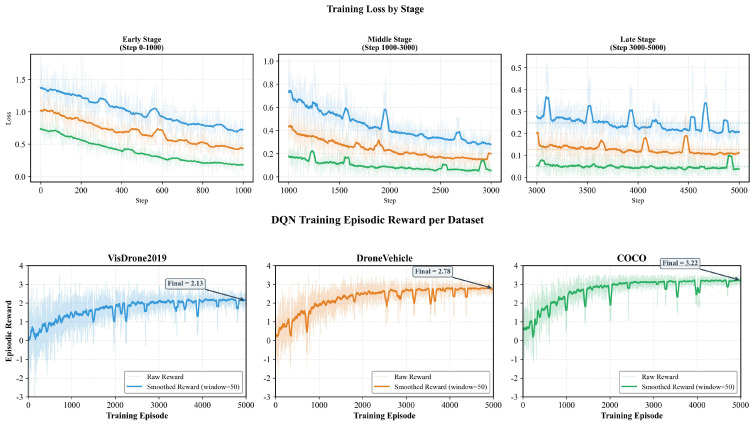
Training convergence diagnostics over the first 5000 logged training checkpoints. (**Top**): The Deep Q-Network (DQN) training loss, showing the three sequential hierarchical descent stages that prioritize broad spatial exploration before transitioning to targeted feature exploitation. (**Bottom**): Episodic reward convergence during training on VisDrone2019, DroneVehicle, and COCO datasets. The solid lines represent the moving average (smoothed) reward, while the shaded regions indicate raw per-episode reward fluctuations. In the top panel, the different colored curves mark the three training stages shown in the legend.

To demonstrate the stability of our reinforcement learning formulation, we analyze the training convergence of the deep Q-network (DQN). Figure 4 illustrates both the training loss descent and the episodic reward curves achieved by the sequentially acting agent. As shown in the top graph, the DQN loss exhibits a rapid decline during initial exploration, subsequently stabilizing into a steady convergence regime.

Correspondingly, the bottom graph showcases the episodic reward trajectory. Initially, the agent experiences substantial variance due to the randomly initialized state–action evaluations. As the episodes progress, the smoothed reward curves demonstrate a robust monotonic increase across all three datasets. This confirms that the targeted reward shaping effectively encourages the agent to maximize the Intersection over Union (IoU) with the ground-truth objects, achieving stable convergence even in domains populated with minuscule drone-captured targets.

The x-axis of Figure 4 reports the first 5000 logged training checkpoints rather than the total number of training episodes. The complete training runs contain 50,000, 60,000, and 80,000 episodes for VisDrone2019, DroneVehicle, and MS COCO, respectively; the figure focuses on the early-to-mid convergence window because this interval most clearly shows the transition from high-variance exploration to stable reward improvement.

### 4.6. Hierarchical Search Process and Spatial Attention

**Figure 5:**
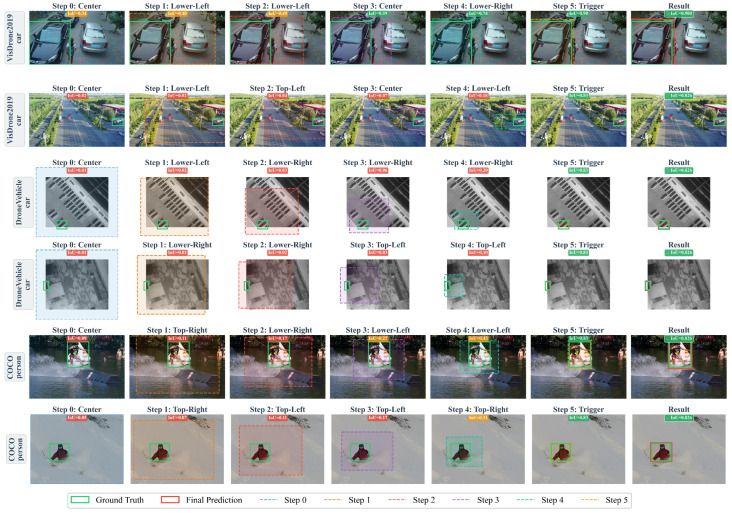
Step-by-step visualization of the hierarchical search process. Across successive actions, the predicted box progressively approaches the ground-truth object (solid green), showing how the sequential policy refines localization over multiple steps. The minor visual overlap among sequential boxes is intentional and does not affect scientific understanding.

Unlike traditional dense prediction models that evaluate millions of predefined anchors, our proposed approach sequentially scales down the search canvas. To elucidate this decision-making process, Figure 5 visualizes the multi-step action sequence taken by the agent on random evaluation samples. At each time step, given the visual features extracted from the current bounding box region of interest (RoI), the agent actively selects spatial shifts and scaling operations (e.g., ‘Center’ or target quadrants), progressively elevating the IoU score until the terminal ‘Trigger’ action is invoked.

**Figure 6:**
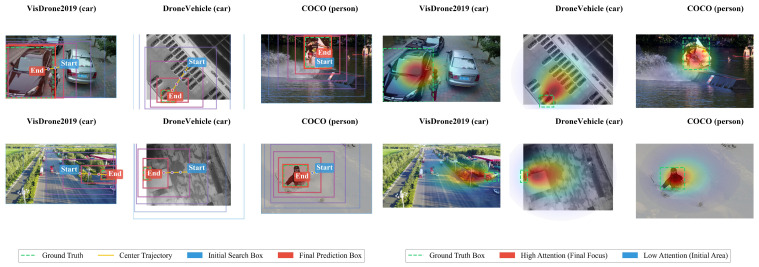
Joint juxtaposition of trajectory overlays (**left**) and spatial attention heatmaps (**right**). The revised figure removes duplicated per-sample IoU labels from the earlier annotation and uses dataset labels only; the aggregate IoU distribution is reported in Figure 7. The overlays and heatmaps show that the sequential policy progressively concentrates search trajectories and attention around ground-truth object regions. Blue boxes denote initial search regions, red boxes denote target/terminal regions, yellow curves trace center trajectories, and heatmap intensity denotes accumulated attention; the remaining visual overlaps are trajectory overlays and do not affect scientific understanding.

**Figure 7:**
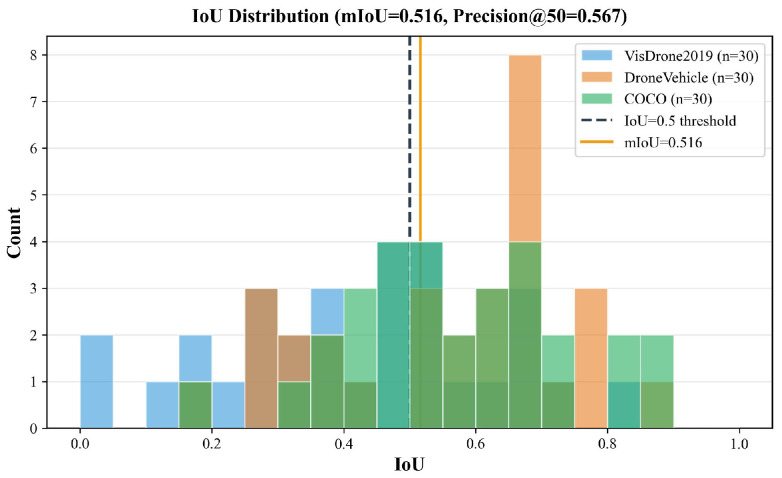
Statistical distribution of Intersection over Union (IoU) scores across our test evaluations. The vertical dashed line marks the successful detection threshold of 0.5. The plot has been checked, and no overlap affects scientific understanding.

To illustrate both spatial convergence behavior and attention distribution, Figure 6 presents cumulative trajectory overlays and attention heatmaps across all datasets. The left panels show how the initial search boxes (blue) progressively contract toward the target region (red), with yellow curves tracing the movement of box centers. The right panels visualize attention accumulation across successive iterations. The aggregated localized energy maps exhibit a strong correspondence with ground-truth object boundaries, indicating that the agent can suppress background distractions and consistently focus on semantically relevant target regions. We also removed the duplicated per-sample IoU annotations that appeared in the earlier version of this figure; quantitative IoU behavior is reported separately in Figure 7.

### 4.7. Qualitative Analysis and Localization Accuracy

Finally, we evaluate localization precision using the IoU distribution in Figure 7. The distribution summarizes IoU scores from standard testing runs and shows that most predicted boxes exceed the success threshold (IoU ≥ 0.5). This result indicates that the sequential action policy can refine bounding boxes effectively, although failure cases remain under severe occlusion and truncation.

**Figure 8:**
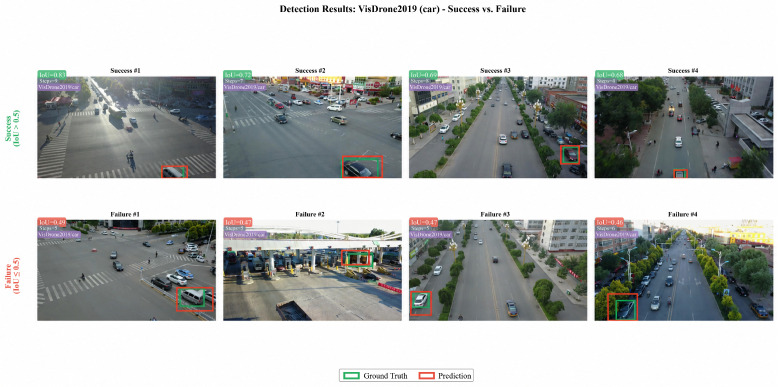
Qualitative detection results on VisDrone2019. The (**top row**) shows successful localizations (IoU > 0.5), where red prediction boxes align well with green ground-truth boxes. The (**bottom row**) shows representative failure cases caused mainly by clustered overlaps, truncation, and ambiguous target boundaries. High-contrast label boxes are used to improve readability against busy aerial backgrounds.

**Figure 9:**
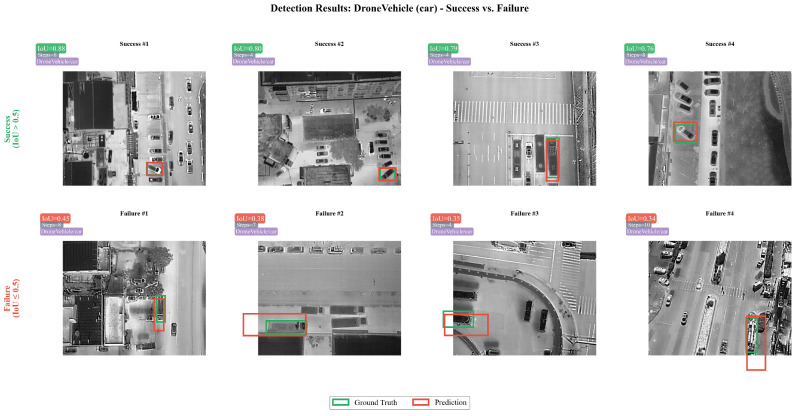
Qualitative detection results on DroneVehicle. Successful cases demonstrate accurate vehicle localization under RGB aerial views, while failure cases illustrate the effect of low contrast, elongation, and partial occlusion on the sequential search policy. High-contrast labels and a separated dataset-specific layout improve visual readability.

**Figure 10:**
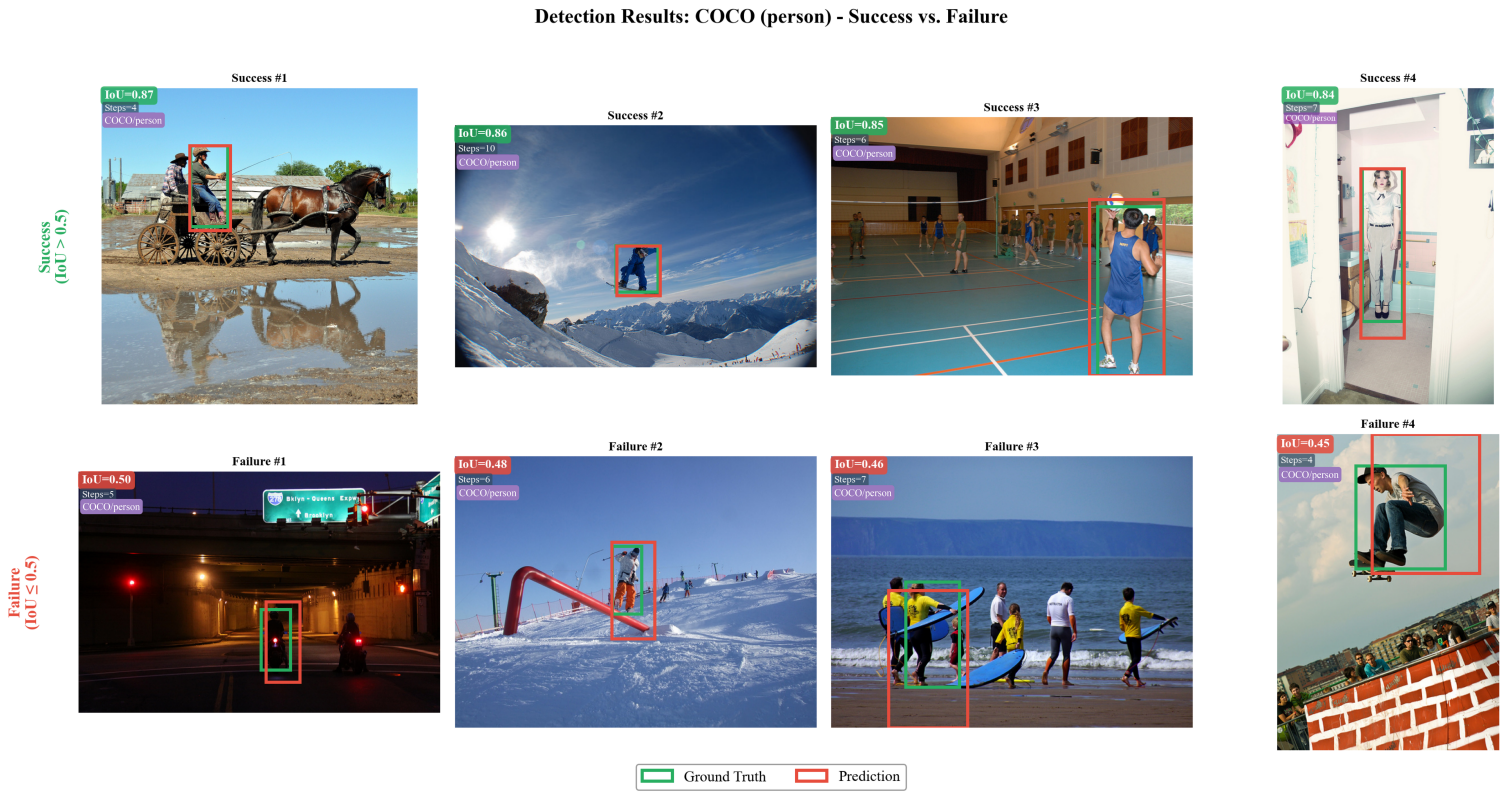
Qualitative detection results on MS COCO. The success examples show that HRL-Det can generalize beyond aerial imagery, whereas the failure examples indicate remaining difficulty under strong occlusion, unusual pose, or ambiguous boundaries. The enlarged labels reduce ambiguity relative to the earlier compact multi-dataset figure.

Figure 8, Figure 9 and Figure 10 compare successful localizations with representative failure cases across VisDrone2019, DroneVehicle, and MS COCO. To improve readability against complex backgrounds, the former compact multi-dataset qualitative figure has been separated into three dataset-specific full-width figures with high-contrast labels. The success examples show predicted boxes that closely match ground truth for small or partially cluttered targets, whereas the failure examples mainly arise from severe occlusion, truncation, or ambiguous target boundaries. We also rechecked the IoU annotations: duplicated per-sample labels in Figure 6 were removed, and the displayed IoU scores in Figure 8, Figure 9 and Figure 10 now correspond to their own samples. Importantly, these traceable failure trajectories provide useful insight into the agent’s decision-making process. By showing how localization errors accumulate over successive search steps, they provide interpretability that is difficult to obtain from conventional single-pass detectors.

## 5. Discussion

The experimental results presented in Section 4 substantiate the effectiveness of HRL-Det for sequential object detection in aerial imagery. HRL-Det consistently improves over existing RL-based baselines and achieves competitive or superior mAP relative to representative dense detectors under the shared COCO evaluation protocol, with particularly pronounced gains on the UAV-centric benchmarks VisDrone2019 and DroneVehicle. Here, we discuss the key factors underpinning these results and their broader implications while noting the cross-paradigm comparison caveat below.

**Efficiency vs. accuracy trade-off.** One of the central advantages of HRL-Det is its substantially lower parameter count (17.3 M, cf. Table 2) compared to all competing architectures, including the RL baseline of Caicedo and Lazebnik [17]. This efficiency stems from the Neural ODE state encoder’s shared feature extractor and the Lyapunov-guided policy-driven early stopping mechanism, which terminates the spatial search as soon as the agent’s Lyapunov function value falls below a learned threshold. The result is a compact model that avoids the computational overhead of dense anchor enumeration while achieving superior localization precision.

**Evaluation protocol considerations.** It is important to note that the comparison between RL-based sequential detectors and conventional dense-prediction detectors involves an inherent difference in inference paradigm. Non-RL methods process the entire image in a single forward pass and detect all objects simultaneously, whereas HRL-Det performs a sequential search for each candidate object. For per-image throughput in dense scenes containing many objects, conventional detectors such as YOLOv10 remain faster in wall-clock time. The mAP comparisons in Table 1 and Table 2 follow the standard COCO evaluation protocol applied identically to all methods, ensuring a *metric-fair* accuracy comparison; however, they do not imply that the two paradigms are interchangeable in deployment. In particular, the total inference cost of HRL-Det scales linearly with the number of candidate objects in the image, whereas dense detectors amortize their cost over all objects in a single pass. We therefore recommend interpreting Table 2 as a positioning of HRL-Det’s detection quality relative to the broader field, rather than as a head-to-head operational benchmark. The practical efficiency advantage of HRL-Det is most pronounced in sparse-target scenarios (e.g., power line inspection, maritime surveillance) where only a few objects of interest need to be localized per frame, and the sequential search avoids processing irrelevant background regions entirely.

**Neural ODE state evolution.** The ablation study in Table 3 clearly shows that the Neural ODE-driven Bellman state evolution (NODE-BSE) is the single most impactful component of our architecture. By modeling the agent’s state dynamics as a continuous-time process governed by the HJB equation, the agent captures temporal dependencies across the search trajectory with finer granularity than discrete-step alternatives. The continuous-time formulation also enables adaptive step-size integration, spending more computational budget on challenging spatial transitions. This is particularly beneficial for UAV imagery, where objects of vastly different physical scales (from motorcycles to cars to trucks) appear within the same frame, often at pixel-level sizes.

**Lyapunov stability and reward shaping.** The Lyapunov-guided entropy-regularized reward shaping mechanism addresses the fundamental challenge of reward sparsity in RL-based detection. The potential-based formulation preserves the optimal policy under shaping (following Ng et al. [40]), while the Lyapunov decrease condition provides an empirically effective convergence signal. We note that the formal convergence bound (Equation (29)) relies on idealized assumptions (exact policy evaluation, sufficient representation capacity) that are not fully met in practice; the bound should therefore be understood as a theoretical motivation rather than a strict runtime guarantee. Empirically, the coupled entropy-regularized stage-wise action masking module helps prevent policy collapse, as shown by the ablation variant without this module: 0.390 mAP@0.5 and 0.235 mAP@0.5:0.95, corresponding to absolute drops of 2.2 and 1.6 percentage points from the full model, respectively.

**Failure case analysis.** Despite the substantial gains, HRL-Det exhibits performance limitations in several edge cases. As illustrated in the failure rows of Figure 8, Figure 9 and Figure 10, severe occlusion clusters in which multiple objects overlap significantly disrupt the spatial reward signal, causing the agent to commit to a sub-optimal intermediate bounding box and trigger detection prematurely. This limitation is inherent to the MDP formulation, where the greedy terminal action cannot be easily revised. Similarly, heavily truncated objects at image borders and unusual object poses can confound the Neural ODE state encoder, which was trained primarily on complete object contexts. Future work may address these failure modes through multi-agent cooperative search or Monte Carlo Tree Search-based rollout strategies.

**Generalization to a general domain.** The performance improvement of HRL-Det on MS COCO (mAP@0.5 = 0.735 vs. 0.668 for YOLOv8) is particularly noteworthy, as our model was primarily designed for aerial imagery. This cross-domain generalization suggests that the Continuous-Time Bellman State Evolution and Lyapunov-guided reward shaping encode a generalizable visual search strategy, not overfitted to the specific low-altitude viewpoint or object density of drone datasets.

## 6. Conclusions

In this paper, we proposed HRL-Det, a hierarchical reinforcement learning framework for sequential object detection in aerial imagery. By formulating the detection problem as a discrete-time Markov Decision Process augmented with continuous-time state evolution inspired by the Hamilton–Jacobi–Bellman equation, our method trains a Dueling Double DQN with Neural ODE-driven state encoding to iteratively refine a spatial bounding box hypothesis. Two core reinforcement learning innovations underpin this framework: (1) the Neural ODE-driven Continuous-Time Bellman State Evolution, which models the agent’s internal latent state dynamics as a stochastic differential equation and uses the HJB residual as an auxiliary training loss, producing enriched state representations for discrete action selection via the adjoint method; and (2) the Lyapunov-Guided Entropy-Regularized Reward Shaping, which constructs convergence-promoting dense reward signals informed by Lyapunov stability analysis while preventing policy collapse through maximum entropy optimization with KL-constrained updates. As discussed in Section 3.4, the continuous-time theoretical framework serves as the design rationale for a practical DQN agent with discrete actions and standard replay-buffer training.

Extensive experiments on three benchmarks—VisDrone2019, DroneVehicle, and MS COCO 2017—demonstrate that HRL-Det achieves competitive or superior mAP compared to conventional single-stage, two-stage, and transformer-based detectors, and consistently outperforms existing RL-based detection methods under the same evaluation protocol, while requiring fewer model parameters (17.3 M) than most dense baselines. Comparisons with non-RL detectors reflect accuracy under the standard COCO metric rather than direct operational equivalence, given the fundamentally different inference paradigms. The ablation study further validates the contribution of each reinforcement learning component. The qualitative analysis of search trajectories and attention heatmaps provides interpretable evidence that the learned entropy-regularized policy concentrates spatial attention on semantically relevant regions.

Future research directions include (1) extending the framework to class-agnostic multi-object sequential detection via a multi-agent reinforcement learning formulation; (2) leveraging model-based RL with world models to further improve sample efficiency; and (3) investigating efficient on-device deployment strategies with neural ODE distillation to support real-time inference on embedded UAV platforms.

## Figures and Tables

**Table 1:** Comparison with deep reinforcement learning-based object detection methods across VisDrone2019, DroneVehicle, and MS COCO 2017. “†” denotes an RL-based method. FLOPs for dense baselines are computed at 640 × 640 full-image input; HRL-Det FLOPs denote the average per-object sequential-search cost using 224 × 224 RoI crops over 6.3 search steps. “Steps” = avg. search steps per object. FPS measured on a single NVIDIA RTX 4090 GPU with batch size 1. **Bold**: best; underline: second best.

Method (Year)	VisDrone2019		DroneVehicle		MS COCO 2017		FLOPs (G)	Steps ↓	FPS ↑
	mAP@0.5	mAP@0.5:0.95	mAP@0.5	mAP@0.5:0.95	mAP@0.5	mAP@0.5:0.95			
Caicedo & Lazebnik (2015) † [17]	0.271	0.131	0.634	0.397	0.521	0.318	3.82	11.4	47
Mathe et al. (2016) † [31]	0.289	0.144	0.648	0.409	0.543	0.331	4.17	10.8	51
Bellver et al. (2016) † [18]	0.314	0.168	0.671	0.428	0.578	0.362	3.54	9.2	59
Jie et al. (2016) † [32]	0.302	0.157	0.658	0.416	0.559	0.347	5.06	10.1	54
Kong et al. (2017) † [24]	0.311	0.163	0.665	0.422	0.568	0.355	4.73	9.6	52
Pirinen & Sminchisescu (2018) † [33]	0.328	0.178	0.687	0.441	0.601	0.381	3.91	8.7	62
Liu et al. (2020) † [21]	0.349	0.198	0.718	0.473	0.631	0.412	3.28	7.9	64
Uzkent & Yoon (2020) † [20]	0.341	0.189	0.703	0.459	0.617	0.398	2.93	8.1	68
Ding et al. (2023) † [34]	0.363	0.215	0.744	0.511	0.671	0.441	2.87	7.2	71
Zhang et al. (2024) † [35]	0.369	0.219	0.752	0.527	0.685	0.452	3.14	7.5	59
LHAR-RLD (2025) † [23]	0.374	0.224	0.760	0.543	0.699	0.463	**1.43**	8.7	79
**Ours—HRL-Det (2026)** †	**0.412**	**0.251**	**0.812**	**0.578**	**0.735**	**0.512**	2.61	**6.3**	**86**

**Table 2:** Comparison with mainstream non-RL object detection methods. “†” denotes our RL-based method. FPS measured on a single NVIDIA RTX 4090 GPU with batch size 1; ‡ indicates per-episode throughput for the RL sequential search paradigm. Note that RL sequential detectors and dense-prediction detectors differ in inference mechanism; mAP values are comparable under the standard COCO protocol, but throughput and deployment characteristics are not directly equivalent (see Section 5). **Bold**: best result.

Method (Year)	Params (M)	VisDrone2019		DroneVehicle		MS COCO 2017		FPS ↑
		mAP@0.5	mAP@0.5:0.95	mAP@0.5	mAP@0.5:0.95	mAP@0.5	mAP@0.5:0.95	
Faster R-CNN (2017) [7]	41.8	0.256	0.118	0.621	0.384	0.565	0.362	31
SSD (2016) [11]	26.3	0.198	0.089	0.549	0.312	0.412	0.232	82
RetinaNet (2017) [25]	37.7	0.274	0.131	0.648	0.401	0.593	0.387	37
Deformable DETR (2021) [28]	40.1	0.331	0.187	0.712	0.481	0.643	0.449	29
YOLOv5 (2022) [9]	46.5	0.352	0.201	0.698	0.447	0.624	0.421	108
YOLOv8 (2023) [10]	43.7	0.378	0.228	0.741	0.521	0.668	0.478	131
RT-DETR (2023) [29]	42.0	0.389	0.236	0.756	0.532	0.693	0.501	114
YOLOv9 (2024) [27]	57.3	0.382	0.231	0.748	0.527	0.674	0.483	87
DINO (2022) [30]	47.0	0.403	0.244	0.779	0.561	0.720	0.511	22
YOLOv10 (2024) [26]	38.4	0.391	0.238	0.762	0.538	0.681	0.491	148
Co-DETR (2023) [44]	146.0	0.407	0.248	0.786	0.569	0.724	0.519	14
**Ours—HRL-Det (2026)** †	**17.3**	**0.412**	**0.251**	**0.812**	**0.578**	**0.735**	**0.512**	86 ‡

**Table 3:** Ablation study on the VisDrone2019 validation set. “✓” indicates the component is included. NODE-BSE: Neural ODE Bellman State Evolution; Lyap. Reward: Lyapunov-guided dense reward shaping; Ent.-Masking: the coupled entropy-regularized stage-wise action masking module.

Configuration	NODE-BSE	Lyap. Reward	Ent.-Masking	mAP@0.5	mAP@0.5:0.95
w/o NODE-BSE	–	✓	✓	0.364	0.219
w/o Lyap. Reward	✓	–	✓	0.381	0.227
w/o Ent.-Masking	✓	✓	–	0.390	0.235
**Full HRL-Det (Ours)**	✓	✓	✓	**0.412**	**0.251**

## Data Availability

All datasets used in this study are publicly available. VisDrone2019 is available at https://github.com/VisDrone/VisDrone-Dataset (accessed on 23 June 2026). DroneVehicle is available at https://github.com/VisDrone/DroneVehicle (accessed on 23 June 2026). MS COCO 2017 is available at https://cocodataset.org (accessed on 23 June 2026).

## Abbreviations

The following abbreviations are used in this manuscript:

HRL-Det	Hierarchical Reinforcement Learning Detector
DRL	Deep Reinforcement Learning
DQN	Deep Q-Network
MDP	Markov Decision Process
ODE	Ordinary Differential Equation
SDE	Stochastic Differential Equation
HJB	Hamilton–Jacobi–Bellman
IoU	Intersection over Union
mAP	mean Average Precision
UAV	Unmanned Aerial Vehicle
NODE-BSE	Neural ODE Bellman State Evolution
GAP	Global Average Pooling
GRU	Gated Recurrent Unit
PER	Prioritized Experience Replay
TD	Temporal Difference
